# Monocytes affect bone mineral density in pre- and postmenopausal women through ribonucleoprotein complex biogenesis by integrative bioinformatics analysis

**DOI:** 10.1038/s41598-019-53843-6

**Published:** 2019-11-21

**Authors:** Kang-Wen Xiao, Jia-Li Li, Zi-Hang Zeng, Zhi-Bo Liu, Zhi-Qiang Hou, Xin Yan, Lin Cai

**Affiliations:** 1 Department of orthopedics, Zhongnan Hospital of Wuhan University, Wuhan, Hubei 430071 The People’s Republic of China; 2 Department of Radiation and Medical Oncology, Zhongnan Hospital of Wuhan University, Wuhan, Hubei 430071 The People’s Republic of China

**Keywords:** Functional clustering, Osteoporosis

## Abstract

Osteoporosis is one of the most common metabolic bone disease among pre- and postmenopausal women. As the precursors of osteoclast cells, circulating monocytes play important role in bone destruction and remodeling. The aim of study is to identify potential key genes and pathways correlated with the pathogenesis of osteoporosis. Then we construct novel estimation model closely linked to the bone mineral density (BMD) with key genes. Weighted gene co-expression network analysis (WGCNA) were conducted by collecting gene data set with 80 samples from gene expression omnibus (GEO) database. Besides, hub genes were identified by series of bioinformatics and machine learning algorithms containing protein-protein interaction (PPI) network, receiver operating characteristic curve and Pearson correlation. The direction of correlation coefficient were performed to screen for gene signatures with high BMD and low BMD. A novel BMD score system was put forward based on gene set variation analysis and logistic regression, which was validated by independent data sets. We identified six modules correlated with BMD. Finally 100 genes were identified as the high bone mineral density signatures while 130 genes were identified as low BMD signatures. Besides, we identified the significant pathway in monocytes: ribonucleoprotein complex biogenesis. What's more, our score validated it successfully.

## Introduction

Osteoporosis is one of the most common metabolic disease affecting thousands of pre- and postmenopausal women^1^. Patients diagnosed with osteoporosis have manifestations such as spine, hip and wrist fracture^2^. Even worse, some patients have enhanced mortality due to bone fracture^3^. Although researchers analyzed the key aspects of osteoporosis, the results were still not comprehensive and thorough^4^. Therefore, finding novel method to deal with osteoporosis is of great significance. There are multiple factors participating in the occurrence and development of osteoporosis by affecting the osteoclast cells, osteoblast cells and regulation of the hormone from endocrine system^5^.

Circulating monocytes, also called peripheral blood monocytes, are bone marrow-derived leukocytes consisting of 3~8% human blood leukocyte^6^, which can further differentiate into many kinds of cells like macrophages, dendritic cells and osteoclast cells^7^. As the precursor cells of osteoclast cells, classic circulating monocytes (CD14++ CD16−) is important for osteogenesis and bone remodeling by producing cytokines (e.g IL-1, IL-6) for osteoclast differentiation, activation and apoptosis^8^. Hence, circulating monocytes are closely related to pathogenesis of osteoporosis, which have been studied for pathophysiology of bone research in the past several years. For instance, Zhang’s study showed that monocytes were related to the postmenopausal osteoporosis in Caucasian female^9^. Monocytes were regarded as an appropriate model to study the pathology of bone in Zhou’s review^10^.

Natural menopause mostly occurs in women between the ages of 40 to 58^11^. Many metabolic and cardiovascular disease are closely related to this stage^12^. Because of the decreased ovarian function, natural menopause is characterized by low estrogen secretion^13^. In recent years, many studies have illustrated the relationship between monocytes and menopause. Phiel’s study demonstrated that differential estrogen receptor expression was detected in monocytes in pre and postmenopausal women^14^. What’s more, estrogen has been found to inhibit RANKL-stimulated osteoclastic differentiation of monocytes in Perrien’s study^15^.

Thousands of genes were involved in the molecular mechanism of interaction between osteoporosis and monocytes^16^, increasing the difficulty of research. Genetic feature screening, enrichment of feature signals, and other bioinformatics methods were used in this study. Weighted gene co-expression network analysis (WGCNA), a comprehensive and novel collection of R package, has been widely used in genomic and bioinformatics study to obtain correlation patterns among genes and detect biomarker or pathway. Unlike the former algorithm differential gene expression analysis, which analyzed difference between samples, WGCNA focuses on relationship between genes and divide them into different modules. Pearson correlation analysis is a method for screening genes that are highly correlated with clinical phenotype. Receiver operating characteristic (ROC) is a method to comprehensively evaluate diagnostic accuracy or discriminate results by combining sensitivity and specificity. After constructing co-expression network, genes with similar expression pattern can be clustered in same module. Then principal component analysis (PCA) were performed on each module to calculate the module eigenvalues. Moreover, the BMD related module were identified by Pearson correlation analysis between module eigenvalues and clinical phenotype. Gene set variation analysis (GSVA), a non parametric, unsupervised method calculating single sample gene set enrichment scores, helping to predict different BMD activation.

In this study, co-expression modules were identified by using 80 monocytes samples from GEO database. Then hub genes highly correlated with BMD were screened by differential gene expression analysis, WGCNA, protein-protein interaction (PPI), Pearson correlation analysis and ROC analysis. Moreover, a novel score system to distinguish the different BMD was constructed, which was validated by independent data sets and k-fold cross-validation. Finally, we identified the ribonucleoprotein complex biogenesis pathway which was significant for pathogenesis of osteoporosis and further provided novel insight for osteoporosis.

## Material and Methods

### Sample collection and data preprocessing

Microarray data sets GSE56815 (https://www.ncbi.nlm.nih.gov/geo/query/acc.cgi?acc=GSE56815), GSE2208^17^, GSE13850^18^ and GSE20941 (https://www.ncbi.nlm.nih.gov/geo/query/acc.cgi?acc=GSE20941) were downloaded from GEO database^19^ at https://www.ncbi.nlm.nih.gov/geo/. The GSE56815 data set was independent training data set while the GSE2208, GSE13850 and GSE20941 were independent test data sets. Then GSE56815, GSE2208, GSE13850 and GSE20941 data sets were further used for analysis and validation. Besides, standardization of raw data by RMA^20^ and z-score methods were performed in the background. The formula of z-score was as follow:

$$z=(x-\mu )/\sigma $$

Z was the standard fraction, x was a specific fraction, μ was the average and σ was the standard deviation.

Moreover each probe was annotated to the genes. Maximum expression value of probe was calculated when genes matched more than one probes.

**Figure 1:**
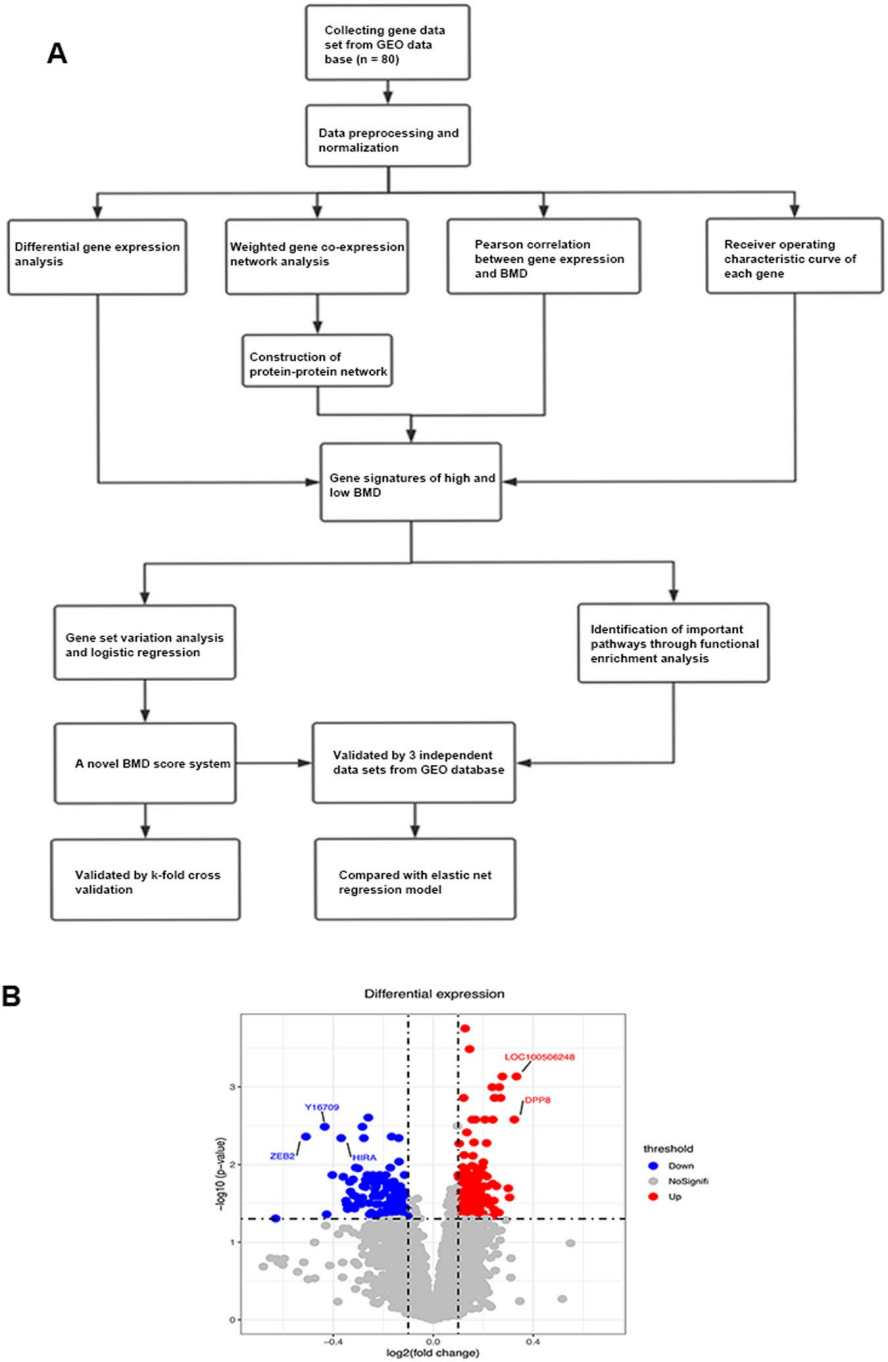
(**A**) Flowchart of this study; (**B**) Differential gene expression analysis for identifying BMD related gene signatures in training data set.

The GSE56815 data set consisted of 80 pre- and postmenopausal caucasian women, 40 with high BMD and 40 with low BMD. The GSE2208 data set included 19 women, 10 with high BMD and 9 with low BMD. The GSE13850 data set consisted of 40 postmenopausal women, 20 with high BMD and 20 with low BMD. The GSE20941 data set consisted of 12 crohn’s disease samples, 6 with osteoporosis and 6 without osteoporosis. Detailed information was shown in Table 1 and Table S14. The flow chart was shown in Fig. 1A.

**Table 1:** The basic information of patients with osteoporosis.

BMD	GSE56815		GSE2208	
	high BMD	low BMD	high BMD	low BMD
postmenopausal	20	20	5	5
premenopausal	20	20	5	4

### Differential gene expression analysis

The training data set (GSE56815) was used for differential gene expression analysis. Differential gene expression were analyzed based on limma packages^21^ in the R language. Differential gene expression analysis was used to find gene signatures of BMD and distinguish the direction of the gene. Gene expression profile data with high dimensional and small sample size features required multiple test controls. The multiple hypothesis test control in this study used the false positive rate control method proposed by Li^22^. Differential gene expression screening criteria: adjust p < 0. 05, fold change (log_2_(FC) ≥ 0.1).

### Elastic net regression model

The GSE56815 data set was used for elastic net regression analysis. Elastic net analysis was performed by using glmnet package^23^ in the R language. For a linear regression model with a sample size of N and a feature dimension of ρ

1 $${{\rm{y}}}_{i}=\mathop{\sum }\limits_{j=1}^{p}\,{\beta }_{j}{x}_{ij}+{\beta }_{0}+\varepsilon $$

Where ε~N (0, σ^2^) is the error term and β_j_ is the regression coefficient of the model, i = 1, 2, …, N, j = 1, 2, …, p.

The least square method with penalty term of elastic network was used to estimate the model parameters, i.e. the regression coefficients beta J and beta o, minimizing the loss function:

2 $$\frac{1}{N}\mathop{\sum }\limits_{i=1}^{N}\,{({y}_{i}-{\beta }_{0}-\mathop{\sum }\limits_{j=1}^{p}{\beta }_{j}x{}_{ij})}^{2}+{{\rm{\lambda }}P}_{\alpha }(\beta )$$

Among them, the penalty items are

3 $${{\rm{P}}}_{\alpha }(\beta )=\alpha ||\,\beta |{|}_{1}+\frac{1-\alpha }{2}||{\beta }_{2}|{|}^{2}=\mathop{\sum }\limits_{j=1}^{p}\,(\alpha |{\beta }_{j}|+\frac{1-\alpha }{2}{{\beta }_{j}}^{2})$$

The penalty items of the elastic network regression method consisted of lasso penalty items ($||\,\beta ||{}_{1}$) and ridge regression penalty items ($\frac{1}{2}||{\beta }_{2}$). The regularization parameter λ in Eq. (2) can adjust the sparsity of the model. The larger the value of λ, the larger the sparsity of the model. The regularization parameter *α* in Eq. (3) adjusted the ratio between ridge regression penalty items and lasso penalty items in the range of λ > 0, *α* ∈ [0, 1], respectively. The Elastic net regression model was shown in Fig. S1.

### Construction of gene co–expression modules network

WGCNA was used for module identification, discovery of phenotype- correlated module, and identification of hub gene. The GSE56815 data set was used for the construction of gene co-expression module network. In this study 13285 genes in 80 samples was used to construct the co-expression network with the R package of “WGCNA”^24^. The Pearson correlation coefficient of two genes was defined as unsigned co-expression similarity. The adjacency matrix was calculated by correlation in power function between two genes and the formula was shown as:

$${{\rm{\alpha }}}_{ij}=|{\rm{cor}}({{\rm{x}}}_{i},{{\rm{x}}}_{j}){|}^{\beta }$$

The power *β* was selected according to standard of approximate scale -free topology and mean connectivity (degree of gene interconnection).

Considering the relationship of genes in the analysis, the adjacency matrix was transformed into the topological matrix. A hierarchical clustering tree^25^ was constructed based on the dissimilarity coefficient between genes. Different branches of the cluster number represented different gene modules and the minimum size of module was 30. Building a clustering tree has two algorithms: static cut tree and dynamic cut tree. Gene modules were correlated to phenotype by calculating the correlation coefficients of the module eigenvectors and the phenotype. Besides, different modules were integrated into one module when the eigenvalue correlation coefficients of different modules were greater than 0.25.

### Identification of clinically significant modules and construction of protein-protein interaction (PPI) network

Module eigengene was identified as first principal component of PCA^26^.

Correlation between modules and phenotype were estimated by using module-trait relationship analysis of WGCNA. We also performed scatter plot of gene significance (GS, the correlation between gene expression and phenotype), the correlation between genes and phenotype and module membership (MM, correlation between gene expression and eigengene of module). Moreover, six modules that were highly correlated with BMD were selected. PPI network was constructed through STRING version.10 and the following protein linkages are presented in a network relationship: interactions from curated databases, experimentally determined interactions, gene neighborhood interactions, gene fusions interactions, gene co-occurrence interactions, textmining interactions, co-expression interactions and protein homology interactions (Interactions between proteins from the same origin)^27,28^. The PPI network was further visualized with cytoscape software^29^. The confidence level for this network was larger than 0.4.

### Analysis of receiver operating characteristic(ROC)

ROC analysis was used for two purposes in the article. First, the ROC analysis was performed to screen genes in WGCNA with AUC greater than 0.7 by using training data set (GSE56815). Besides, ROC analysis was also used for evaluation of predictive value in logistic regression by using both test data sets (GSE2208, GSE13850, GSE20941) and training data set (GSE56815). The horizontal axis was 1- specificity while the vertical axis was sensitivity. The ROC curve was based on a series of different two-category methods (demarcation value or decision threshold). Each point on the curve corresponded to a different threshold. the abscissa represented the continuous gene expression value while the ordinate represented the 0 and 1 (0 represented negative data sets while 1 represented positive data sets). The positive data sets represented high bone mineral density while the negative data sets represented low bone mineral density. Then we calculated the area under curve (AUC) and screened for genes with an AUC greater than 0.7. The ROC analysis was performed by the pROC package^30^ in R language.

### Gene set variation analysis

The GSVA package^31^ was installed to perform gene set variation analysis. We used the expression data of selected genes from modules. Then a sequenced gene list was formed according to the differential gene expression analysis of the high BMD and low BMD. The identified high BMD and low BMD gene signatures were divided into two novel gene lists. The single sample ES of two novel lists were calculated by GSVA. The formula was as follows:

$${{\rm{ES}}}_{jk}^{diff}=|E{S}_{jk}^{+}|-|ES{\,}_{jk}^{-}|$$

The j represented sample while k represented gene set. The ESjk+ represented the largest positive random walk deviations, conversely the ESjk− represented the largest negative result. Finally a novel BMD model was put forward by integrating these two ES values based on logistic regression.

### Gene set enrichment analysis

Gene ontology (GO)^32^ and kyoto encyclopedia of genes and genomes (KEGG)^33^ enrichment analysis was performed by using for visualization and annotation (www.webgestalt.org)^34^. Gene set enrichment analysis (GSEA)^35^ was further performed by calculating the enrichment score (ES) and estimating the significance of ES. Finally a multiple hypothesis test was performed to calculate the false positive discovery rate (FDR). The overall analysis was performed under the situation of  p < 0.05 and FDR < 0.05.

### K-fold cross-validation

K-fold cross-validation is a widely used method for predictive error estimation under limited samples^36^. Specifically, training data set GSE56815 is divided into k disjoint subsets (blocks) with approximately the same capacity (in this article k = 3). The capacity = n/k. Training data set GSE56815 was randomly divided into 3 parts. Any two parts of the GSE56815 were used as training sets while the rest was used as test data set. Therefore three training data sets and three corresponding test data sets were prepared for further analysis.

## Results

### Raw data collection and hierarchical clustering analysis

Raw gene expression data of monocytes in osteoporosis were downloaded from the GEO database (http://www.ncbi.nlm.gov/geo) with the accession of GSE56815 and GSE2208, containing 80 and 19 samples, respectively. The microarray platform of these two data sets and GSE13850 was GPL96. Besides, the microarray platform of GSE20941 was GPL 4133. Finally we collected 13285 genes and performed the hierarchical clustering analysis.

### Differential gene expression analysis

The training data set (GSE56815) was used for differential gene expression analysis. Differential gene expression analysis was used to identify BMD related gene signatures based on different BMD. The results of differential gene expression analysis were shown in the Fig. 1B. Gene ZEB2 (log_2_(FC) = 0.33, adjust p = 0.00073), Y16709 (log_2_(FC) = −0.43, adjust p = 0.0032), HIRA (log_2_(FC) = −0.37, adjust p = 0.0045), LOC100506248 (log_2_(FC) = 0.33, adjust p = 0.00073) and DPP8 (log_2_(FC) = 0.32, adjust p = 0.0026) were top significant differential expression genes.

### Construction of gene co–expression module

**Figure 2:**
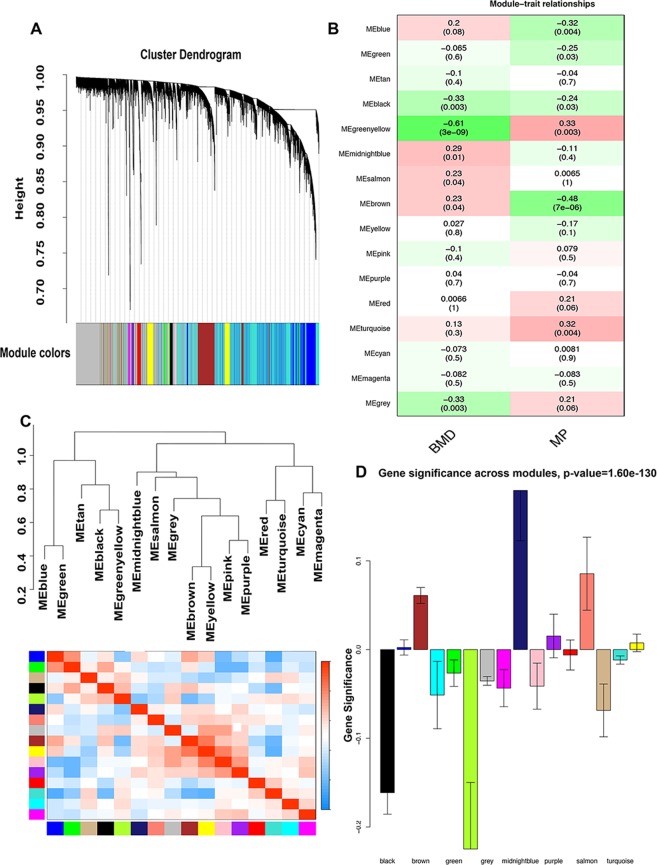
Process of WGCNA. (**A**) dynamic tree cut based on 1- TOM; (**B**) heat map of the correlation between the module eigenvalue and the BMD phenotype and menopausal phenotype; (**C**) clustering heat map of module eigenvalue; (**D**) Analysis of GS across modules.

The co-expression modules were constructed with expression data of genes from GSE56815 data set. Then we selected the appropriate power value as 10 due to the signed R^2 of scale free topology model was 0.9 and the mean connectivity was relatively lower (Fig. S2). As shown in Fig. 2A, 16 modules were identified with dynamic tree cut based on 1- topological overlap matrix (TOM). Here are the number of genes in each module: 4560 (module turquoise), 3704 (module grey), 1772 (module blue), 1389 (module brown), 593 (module yellow), 302 (module green), 210 (module red), 158 (module black), 145 (module pink),105 (module magenta), 88 (module purple), 69 (module green yellow), 67 (module tan), 60 (module salmon), 32 (module cyan),31 (module midnight blue). The average gene number of each module is 830.

### Analysis of model-trait relationship and identification of significant modules

The first principal component was defined as module eigenvalue. The heat map of the correlation between the module eigenvalue and the BMD phenotype was shown in Fig. 2B. Six modules highly correlated with BMD phenotype were selected: module black (cor = −0.33, p = 0.003), module green yellow (cor = −0.61, p = 3e-09), module midnight blue (cor = 0.29, p = 0.01), module salmon (cor = 0.23, p = 0.04), module brown (cor = 0.23, p = 0.04), module grey (cor = −0.33, p = 0.003). Then hierarchical clustering analysis was performed on module eigenvalue and then 16 modules were separated into two clusters, consisting of 5 modules and 11 modules, respectively. Besides, module black and module green yellow had higher interaction connectivity with each other. Clustering heat map of module eigenvalue was shown in Fig. 2C.Then we performed scatter plot of GS and MM for six modules (Fig. S3). Module midnight blue and module salmon showed high correlation with BMD phenotype (module midnight blue cor = 0.14 and p = 0.45, module salmon cor = 0.37 and p = 0.0036, module black cor = 0.11, p = 0.17, module green yellow cor = 0.69, p = 5.5e-11, module brown cor = 0.13, p = 1.2e-06, module grey cor = 0.16, p = 1.2e-22). Analysis of GS across modules were further performed (Fig. 2D). Module green yellow and module midnight blue showed higher GS while module blue showed the lowest significance. Finally six modules (black, green yellow, midnight blue, salmon, brown and gray) were selected for further analysis.

### Construction of protein-protein interaction (PPI) network

**Figure 3:**
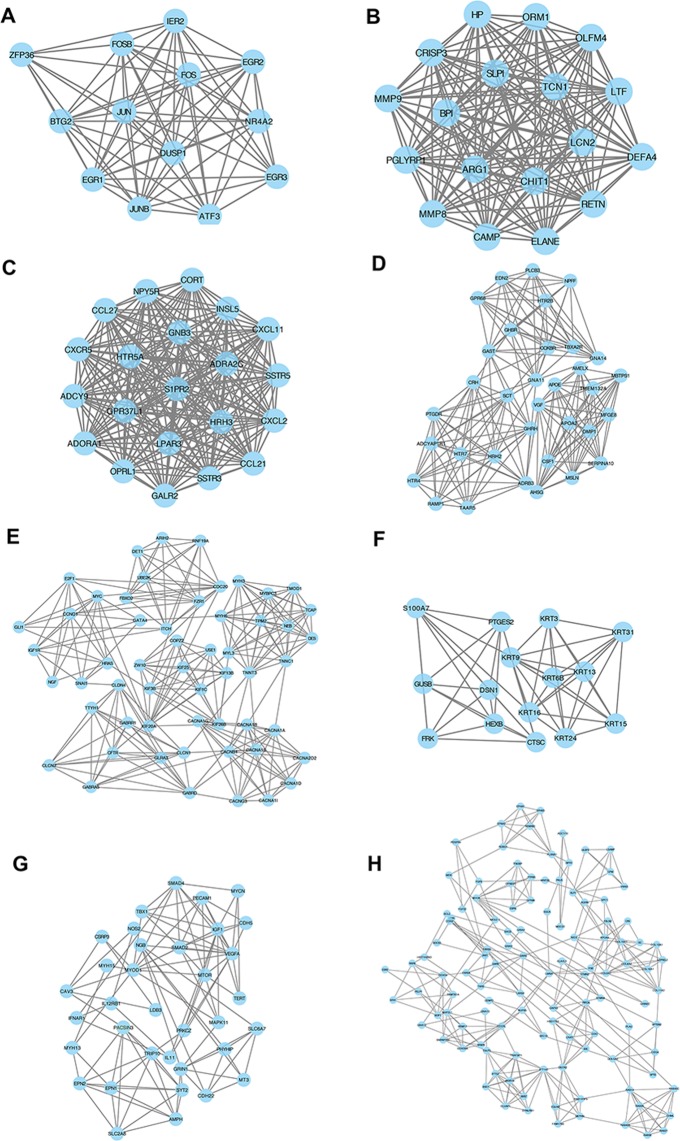
PPI network of modules. (**A**) sub network of midnight blue; (**B**) sub network of salmon; (**C**–**E**): sub network of brown.

We constructed PPI network for five modules separately except module gray due to its low connectivity. The sub network of five modules were displayed in Fig. 3 while the main network of five modules were shown in Fig. S4–S8. Several sub network were identified from module brown, salmon and midnight blue by molecular complex detection tool. The function analysis of proteins from these modules and the results were shown in the Table S1–13. The protein from module black showed high ES with GO:1900371 regulation of purine nucleotide biosynthetic process ( p = 2.09E-06, FDR = 0.010) while the protein from module green yellow showed GO:0071383: cellular response to steroid hormone stimulus ( p = 7.36E-05, FDR = 0.039), GO: 0002283 neutrophil activation involved in immune response ( p = 8.29E-08, FDR = 1.95E-04). Besides, GO:0032870: cellular response to hormone stimulus (p = 6.60E-06, FDR = 5.22E-04) and GO: 1901700 response to oxygen containing compound (p = 3.95E-12, FDR = 1.80E-08) were identified in module midnight blue. The sub network of midnight blue exhibited GO:0009719 response to endogenous stimulus (p = 6.74E-12, FDR = 5.23E-08) and GO: 0009725 response to hormone (p = 9.57E-11, FDR = 2.18E-07). Moreover, GO:0002446: neutrophil mediated immunity (p = 0, FDR = 0) and GO: 0006955 immune response (p = 0, FDR = 0) were presented in module salmon and its sub network, respectively. Besides, GO:0046883: regulation of hormone secretion (p = 7.57E-05, FDR = 0.0051), GO: 1990869 cellular response to chemokine (p = 5.06E-06,FDR = 0.0014), GO:0019932 second-messenger-mediated signaling (p = 5.36E-10, FDR = 9.74E-07), GO: 0034762 regulation of transmembrane transport (p = 9.90E-08, FDR = 3.00E-05), GO: 0001775 cell activation (p = 7.54E-05, FDR = 0.023), GO: 0071363 cellular response to growth factor stimulus (p = 4.89E-06, FDR = 6.99E-04) and GO: intracellular transport (p = 1.08E-08, FDR = 9.16E-05) were displayed in module brown and its six sub network, respectively. In these biological processes, GO: 0009725 response to hormone were closely related to effect of estrogen, which was main treatment for pre- and postmenopausal women. What’s more, GO:1900371 regulation of purine nucleotide biosynthetic process was found in module black. The analysis of hub gene (degree > 2) because of the strategy of class I error reduction in network of each module were further performed.

### Receiver operating characteristic analysis

**Figure 4:**
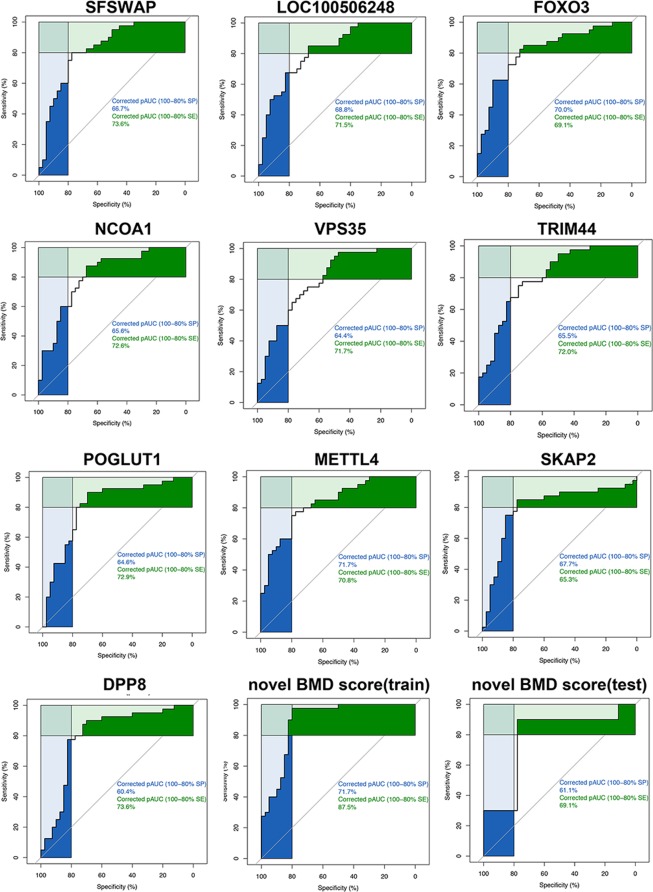
ROC analysis of genes and novel BMD score.

ROC analysis was performed for all genes from six modules (midnight blue, green yellow, salmon, brown, grey, black), respectively. The ROC curves of genes with top 10 AUC (SFSWAP, LOC100506248, FOXO3, NCOA1, VPS35, TRIM44, POGLUT1, METTL4, SKAP2 and DPP8) were further displayed in the Fig. 4. The ROC curve of SFSWAP showed the highest AUC value = 0.831. Besides, gene LOC1005062 exhibited AUC value = 0.831. Gene FOXO3 was found with AUC value = 0.826. NCOA1 was displayed with AUC value = 0.821. Moreover, gene VPS35 showed AUC value = 0.821. What’s more, AUC value = 0.819 were exhibited in gene TRIM44. Gene POGLUT1 manifested AUC value = 0.816. Gene METTL4, a significant factor for DNA methylation, displayed AUC = 0.815 while gene SKAP2 showed AUC value = 0.812. Finally DPP8 was found with AUC value = 0.811. 236 genes were identified for subsequent analysis based on the AUC value > 0.7.

### Construction of novel BMD model and validation

230 genes were further identified from modules based on the area under curve (AUC) value > 0.7, hub gene selection through PPI network and Pearson correlation between BMD and genes. Compared with the osteoporosis-related database^37^, 33 genes belonged to the osteoporosis-related gene family and 11 genes had been reported as osteoporosis-related genes (ADAM17, ALOX12B, CAMKK2, ELN, HAMP, MITF, PTPN1, SOCS3, TNFSF11, VHL, VPS35). Nearly 20% of genes were associated with osteoporosis. Moreover, due to differential gene expression in high and low BMD, these genes were divided into two groups: high BMD group (100 genes) and low BMD group (130 genes). What’s more, we constructed novel high BMD score and low BMD score based on these two groups. The novel high BMD score was positively related to BMD with cor = 0.674, p = 7.336E-12 while low BMD score was negatively related to BMD with cor = −0.670, p = 1.098E-11. To further integrate the above two scores, logistic regression was used to create a unified score system. The formula was as follows:

$$Novel\,BMD\,score=106.929\ast low\,BMD\,score+102.993\ast high\,BMD\,score$$

**Figure 5:**
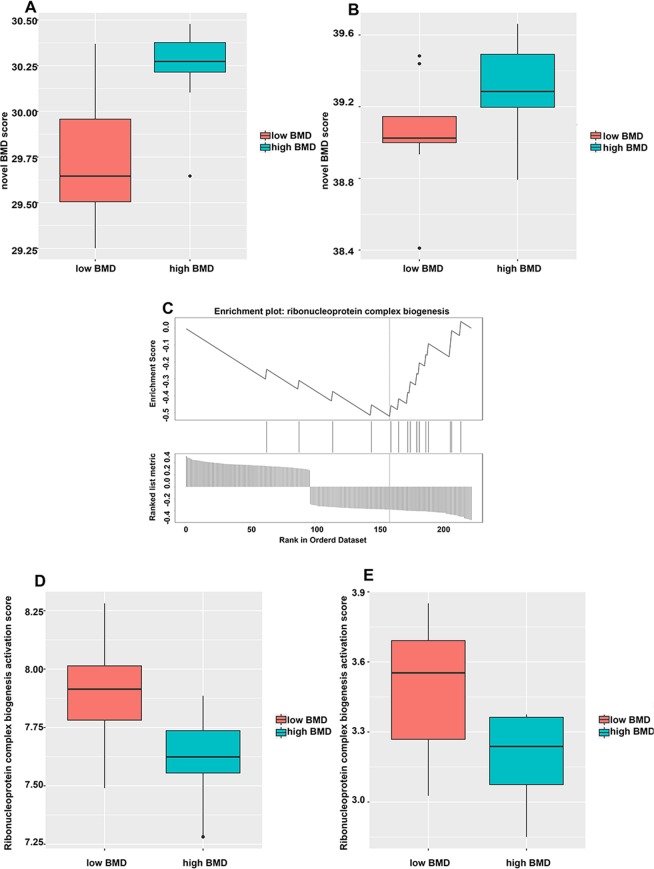
GSEA analysis and box plot. (**A**) box plot of training data; (**B**) box plot of validation data; (**C**) GSEA analysis of 230 BMD related genes; (**D**) box plot of ribonucleoprotein complex biogenesis activity and BMD in training data; (**E**) box plot of ribonucleoprotein complex biogenesis activity and BMD in validation data.

In order to test the robustness of results of our novel BMD model, k-fold cross-validation was performed. The ROC analysis and Pearson correlation analysis were performed to evaluate the predictive value of the model. The results were shown in the Fig. S11–16 below. In training data set 1, the AUC = 0.82 with cor = 0.53 while in test data set 1 the AUC = 0.93 with the cor = 0.70. In training data set 2 the AUC = 0.54 with cor = 0.55 while in test data set 2 the AUC = 0.91 with cor = 0.69. In training data set 3 the AUC = 0.92 with the cor = 0.69 while in test data set 3 the AUC = 0.76 with the cor = 0.44. Besides, independent test data sets GSE13850 and GSE20941 were used to validate our novel BMD score. The results were shown in the Fig. S17 –18. In test data set GSE13850 the AUC = 0.61 while in test data set GSE20941 the AUC = 0.94 with cor = 0.71. Afterward, we verified it from test data set with the accession of GSE2208 with 19 samples in the GEO database. Besides, ROC analysis was performed in training data set GSE56815 as well as test data set GSE2208 and the results were shown in the Fig. 4. The training data (GSE56815) showed AUC = 0.890 while in the test data (GSE2208) the AUC = 0.7778. Moreover, box plot for these two data sets were presented in the Fig. 5A,B. The results showed both the training data set (GSE56815) and test data set (GSE2208) distinguished well between high and low BMD groups. The correlation of training data set (GSE56815) and test data set (GSE2208) were calculated by Pearson correlation, respectively. The results showed cor = 0.724, p = 3.057E-14 in training data set (GSE56815) while cor = 0.428, p = 0.068 in test data set (GSE2208). The p was also calculated by students' t-test with the results of p = 1.009E-12 in training data set (GSE56815) and p = 0.072 in test data set (GSE2208). We also collected smoking-related osteoporosis samples from GSE13850. Our novel BMD score dropped significantly in smoking patients with osteoporosis compared with non-smoking patients with osteoporosis (p < 0.05). The above results proved this novel BMD score could predict BMD effectively.

### Ribonucleoprotein complex biogenesis: a significant pathway to BMD

**Figure 6:**
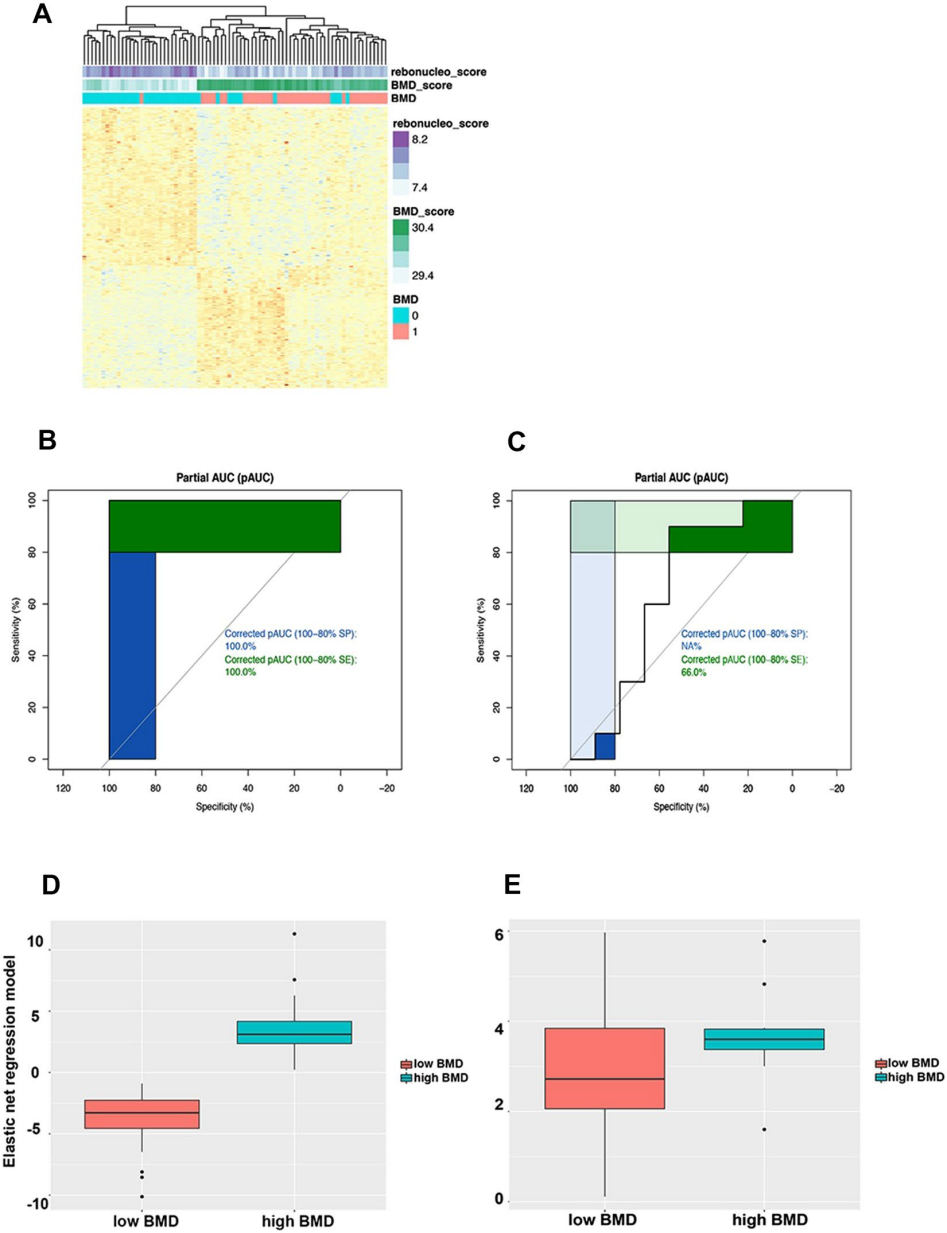
Gene clustering heat map analysis, ROC analysis of genes and elastic net regression network and box plot. (**A**) Gene clustering heat map analysis; (**B**,**C**) ROC curve of training data set (GSE56815) and validation data set (GSE2208) with elastic net regression model; (**D**,**E**) Box plot for training data set (GSE56815) and validation data set (GSE2208).

These 230 genes were used to perform gene set enrichment analysis and the results showed that genes were only significantly enriched in GO: 0022613 ribonucleoprotein complex biogenesis with the ES = −0.520 (p = 0.0035, FDR = 0.040, Fig. 5C). GSVA analysis was further performed among 11 genes (SFSWAP, NVL, RPL3, UTP6, PTEN, DIS3, EIF2D, GTF3A, NOP16, RPLP0, EIF3F) belonging to ribonucleoprotein complex biogenesis. High ribonucleoprotein complex biogenesis activity was correlated with low BMD (cor = −0.634, p = 2.271e-10, Fig. 5D). Independent test data set GSE2208 was used for validation. Since there were only 5 of the 11 ribonucleoprotein complex biogenesis genes matched the probe platform, all genes belonging to ribonucleoprotein complex biogenesis were included form GEO database and intersected with gene expression to obtain 254 genes. The same activity of ribonucleoprotein complex biogenesis was obtained by the same GSVA analysis as the training method (cor = −0.55, p = 0.014, Fig. 5E). Finally gene hierarchical clustering heat map were shown in Fig. 6A.

### Elastic net regression model

In order to compare with our novel model, the elastic net regression model were performed on training data set and test data set, respectively. The training data set (GSE56815) exhibited excellent results (AUC = 1.00, cor with BMD = 0.87, p < 2.2e-16) while the test data set (GSE2208) showed unsatisfied results(AUC = 0.63 with cor = 0.25, p = 0.2866). The ROC curve and box plot for training data set (GSE56815) and test data set (GSE2208) were displayed in Fig. 6B–E.

## Discussion

The novel model and work flow were put forward by integrating WGCNA and GSVA to identify the relationship between circulating monocytes and BMD.

Osteoporosis is one of the most common metabolic disease in the world. Circulating monocytes are precursor cells of osteoclasts, which are essential to the bone destruction and remodeling. Previous study concentrated on proteomics of osteoporosis in postmenopausal women and revealed the potential individual key genes and pathway to the osteoporosis. The pathway showed BMD was associated with arrhythomogenic right ventricular cardiomyopathy, translocation of GLUT4 to the plasma membrane, tight junction, cell-cell communication and platelet degranulation^38^. However, the validation of key genes by independent samples were not used and the research methods were not comprehensive enough. Therefore, in this research differential gene expression analysis was performed. The GSEA results for differential gene expression analysis were shown in Fig. S9. The results turned out that HALLMARK_ESTROGEN_RESPONSE_EARLY: early estrogen response was the significant pathway. Estrogen replacement therapy was proved to greatly improve bone mineral density and prevent bone fracture^39^. Co-expression modules were constructed by WGCNA. Then six modules were further identified and PPI net work was constructed to identify hub genes. Besides, gene set enrichment analysis and gene set variation analysis were performed. Based on the GSEA results for each module, GO:1900371: regulation of purine nucleotide biosynthetic process was the significant pathway in module black. Previous study showed that extracellular nucleotides played a important role in osteoblast function by signaling through P2 receptors^40^. Genes in module green yellow were enriched in GO:0071383: cellular response to steroid hormone stimulus. Previous study showed that estrogen, a steroid hormone could regulate bone mineral density^41^. In module midnight blue, GO:0032870: cellular response to hormone stimulus was the significant pathway. Recent study showed that estrogen loss could cause osteoporosis^42^. In module salmon, GO:0002446: neutrophil mediated immunity was the significant pathway. Recent study showed that neutrophils could upregulate the expression of RANKL, which could induce the osteoclastogenesis and regulate the bone mineral density^43^. Genes in module brown were enriched in GO:0046883: regulation of hormone secretion. Recent study showed that osteoblast development would be increased by interleukin-6 after estrogen loss^44^. Moreover a novel BMD score system was constructed, which showed a significant predictive effect on BMD. According to the results of WGCNA, six modules (module black, module green yellow, module midnight blue, module salmon, module brown, module grey) were significantly associated with BMD. Besides, PPI network were constructed to further identify hub genes. ROC curve of genes with top 10 AUC were displayed. Gene FOXO3 was closely related to the oocyte maturation and ovulation from ovarian follicle, which could be a potential factor affecting menstruation and estrogen^45^. Moreover NCOA1 was shown to be a key factor for the signaling pathway of estrogen^46^. Finally 230 genes highly correlated with BMD were identified based on ROC, PPI network and Pearson correlation analysis. 100 genes were associated with high BMD and 130 genes were associated with low BMD. The GO and KEGG enrichment analysis showed that the term GO:0032870 (cellular response to hormone stimulus) was important for the treatment of pre and postmenopausal women since many kinds of drugs like estrogen and hormone replacement drugs had been already approved for marketing^47^, playing an important role in the regulation of menstrual cycle and the development of puberty and secondary female sex characteristics^48^. The result of GSEA showed that genes were only enriched in GO: 0022613 ribonucleoprotein complex biogenesis. Gene PTEN and gene RPL3 were identified as the significant genes in ribonucleoprotein complex biogenesis pathway from our study. Previous study proved that the PTEN tumor suppressor inhibits telomerase activity by decreasing hTERT mRNA levels^49^. Besides, gene RPL3 could regulate the telomerase activity^50^. Telomerase activity is closely related to bone mineral density and osteoporosis. Former study showed that mutational inactivation of the gene WRN and gene TERC (encoding the telomerase RNA component) would lead to telomere dysfunction and cause osteoporosis with low cortical bone mineral density^51^. Moreover, the expression of telomerase would increase the bone formation *in vivo*^52^. What’s more, telomerase would accelerate the osteogenic differentiation of mesenchymal stem cells^53^. The schematic was shown in Fig. S10. In recent study heterogeneous nuclear ribonucleoprotein L was found to restrained osteogenic differentiation of periodontal ligament stem cells^54^. What’s more, small nuclear ribonucleoprotein polypeptide N was discovered to promote osteogenic differentiation of bone marrow mesenchymal stem cells^55^. The high activity of ribonucleoprotein complex biogenesis was highly correlated with low BMD, which was further validated by independent data set. These findings may provide some new insights on the study of monocytes and bone mineral density. The elastic net regression model was also performed to predict the BMD, However, due to the overfitting in training data set, this method was not suitable in this study. Our approach was not based on regression coefficients, but on the expression of specific genetic features, which could reduce overfitting. Moreover, we are looking forward to more research to prove our findings because of the limitation of the sample size.

## Conclusion

In general, a novel score system which was able to predict the BMD was constructed. Moreover, the ribonucleoprotein complex biogenesis pathway were identified as key part of occurrence and development of osteoporosis.

## Supplementary information

Supplementary information

Table S1~S14

## Data Availability

The datasets analysed during the current study are available in the GEO datasets (https://www.ncbi.nlm.nih.gov/).
