# Supplementary material for: Monocytes affect bone mineral density in pre- and postmenopausal women through ribonucleoprotein complex biogenesis by integrative bioinformatics analysis
Source: Sci Rep. 2019 Nov 21;9:17290. doi: 10.1038/s41598-019-53843-6 (PMC6872746; doi:10.1038/s41598-019-53843-6)
Supplement: Supplementary file 1 — Supplementary information [file 41598_2019_53843_MOESM1_ESM.docx]

**Monocytes affect bone mineral density in pre- and postmenopausal women through ribonucleoprotein complex biogenesis by integrative bioinformatics analysis**

Kang-Wen, Xiao^1^; Jia-Li, Li^2^; Zi-Hang, Zeng^2^; Zhi-Bo, Liu^1^; Zhi-Qiang, Hou^1^; Xin Yan^1^; Lin Cai^1,*^

1. Department of orthopedics, Zhongnan Hospital of Wuhan University, Wuhan, Hubei, 430071, The People's Republic of China
2. Department of Radiation and Medical Oncology, Zhongnan Hospital of Wuhan University, Wuhan, Hubei, 430071, The People's Republic of China

*Correspondence author: Lin Cai. Email: orthopedics@whu.edu.cn. Tel:+86 1388609 6467

Key words: monocytes, WGCNA, GSVA, osteoporosis, bone mineral density, ribonucleoprotein complex biogenesis


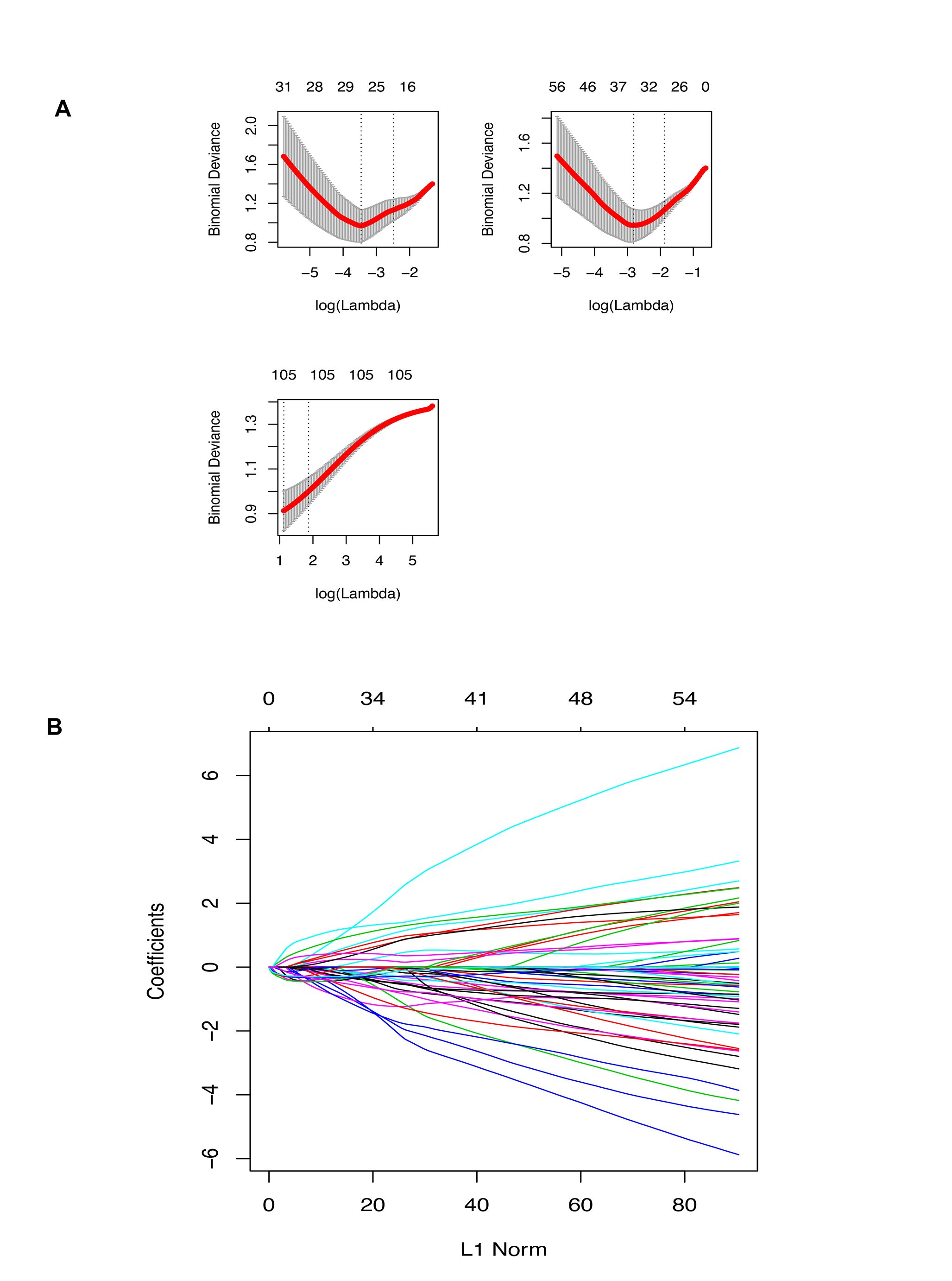


Fig.S1 Elastic net regression model


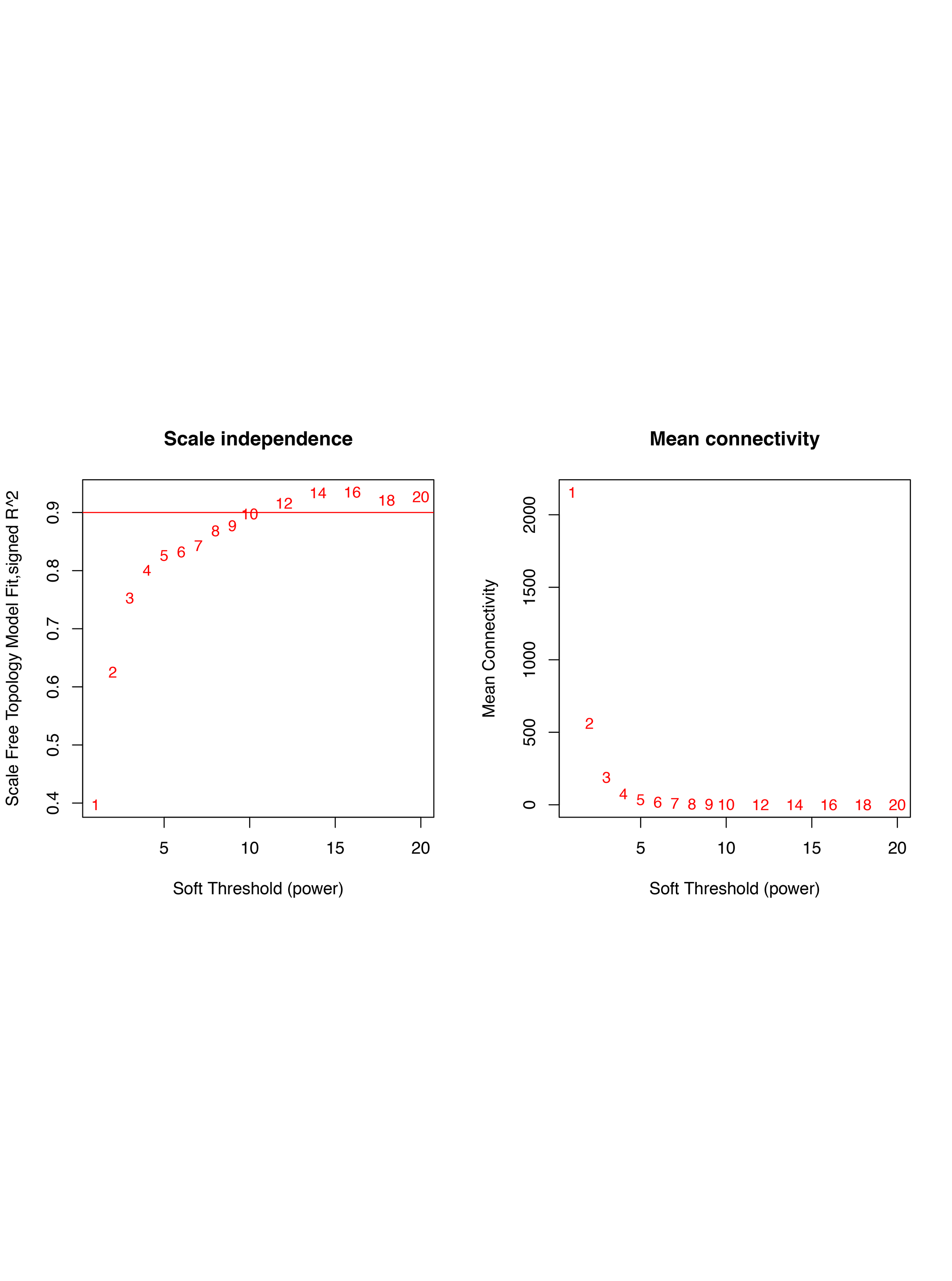


Fig.S2 Power selection plot


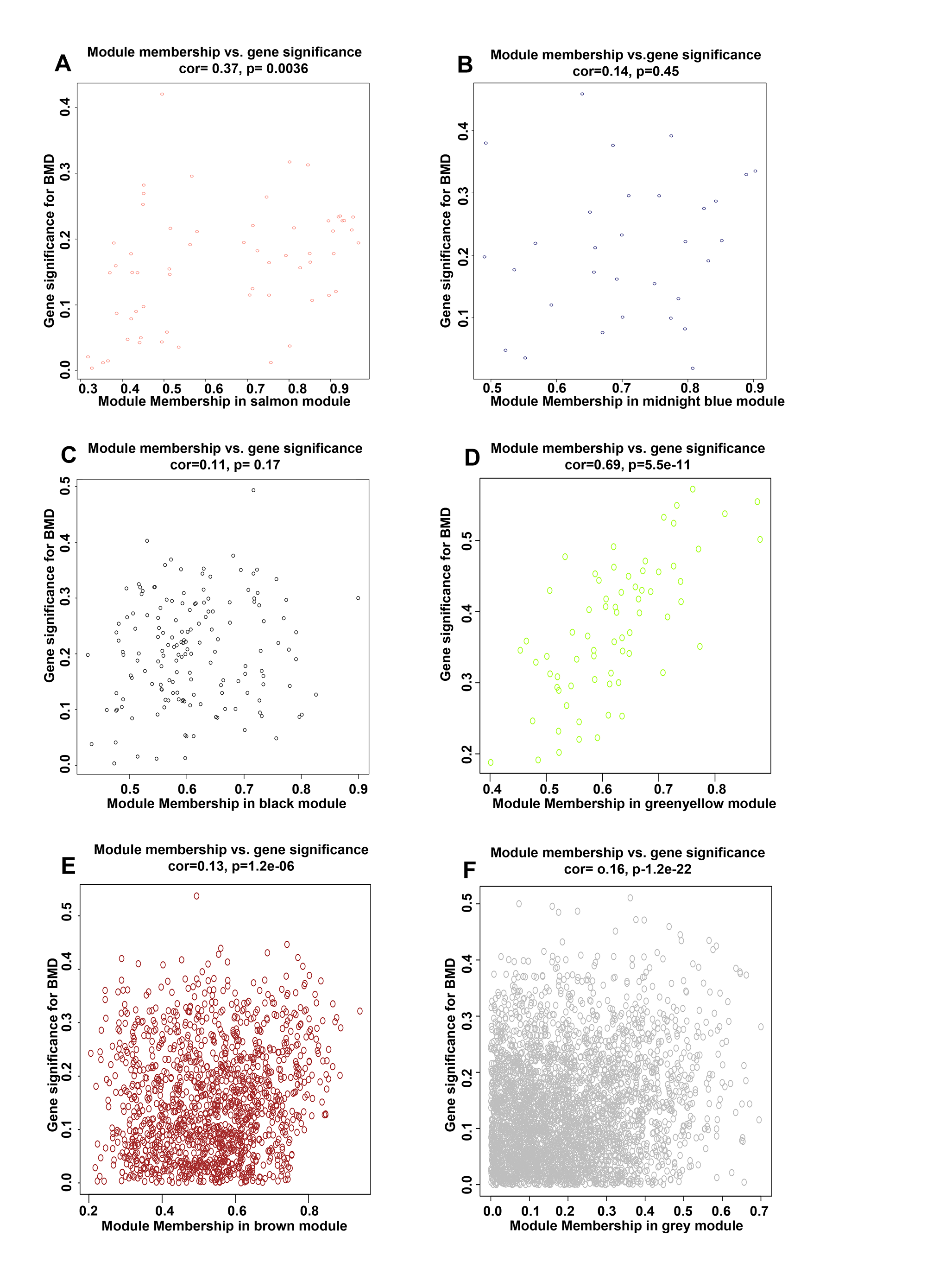


FigureS3. Scatter plot of six modules. Scatter plots for salmon(A),Midnight blue(B), black(C), green yellow(D),brown(E), grey(F), respectively


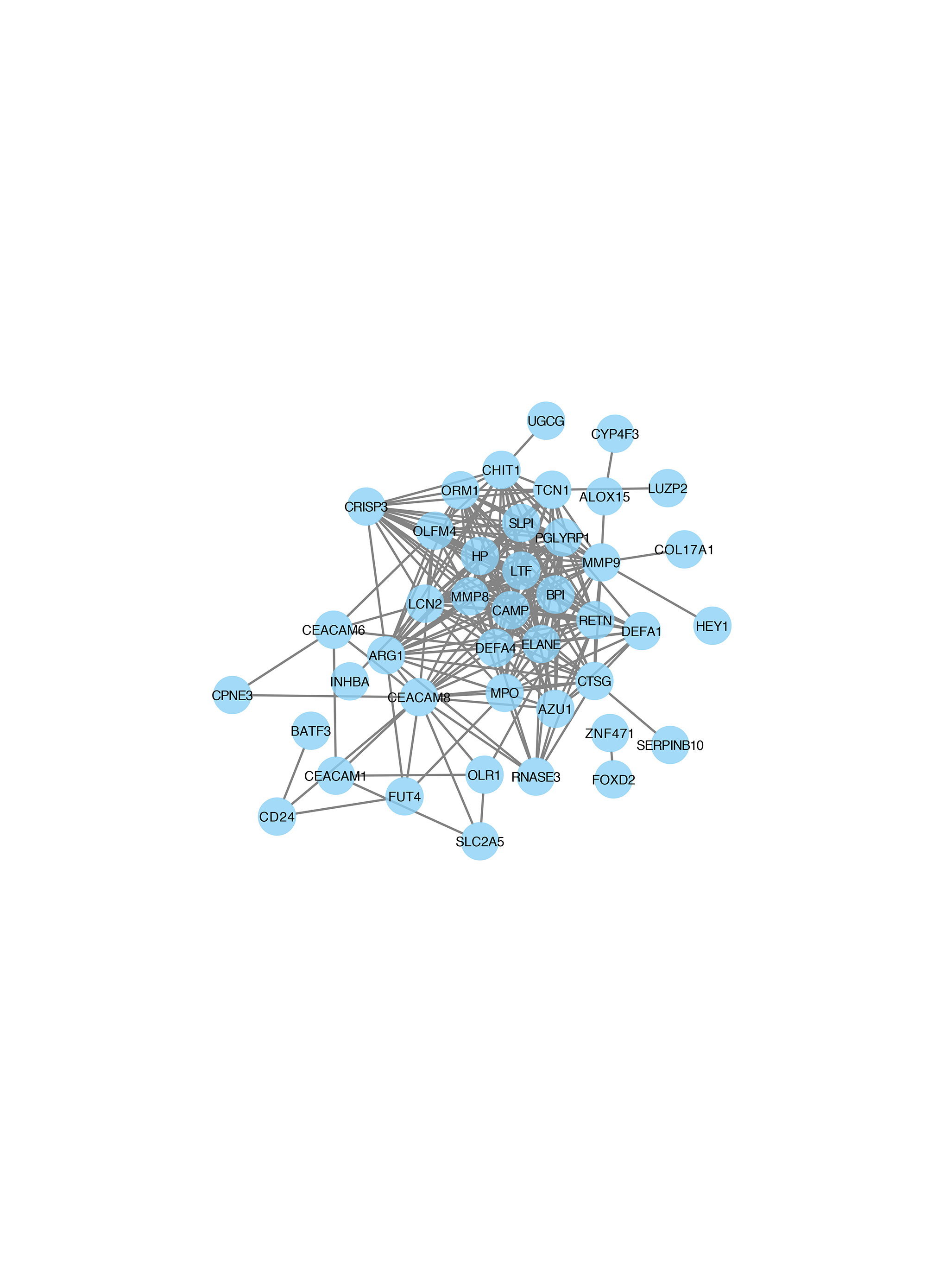


FigureS4. PPI network of module salmon


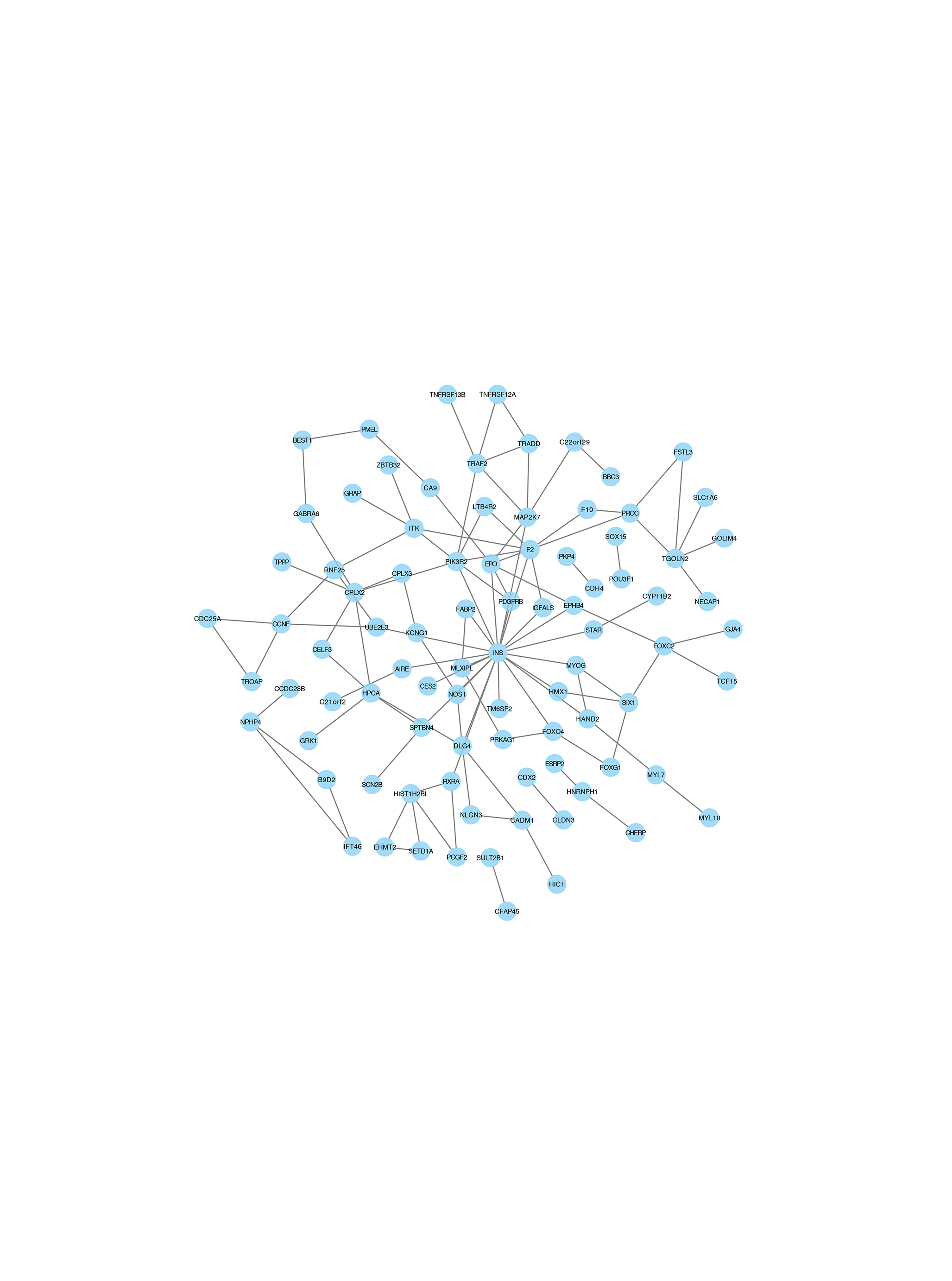


FigureS5. PPI network of module black


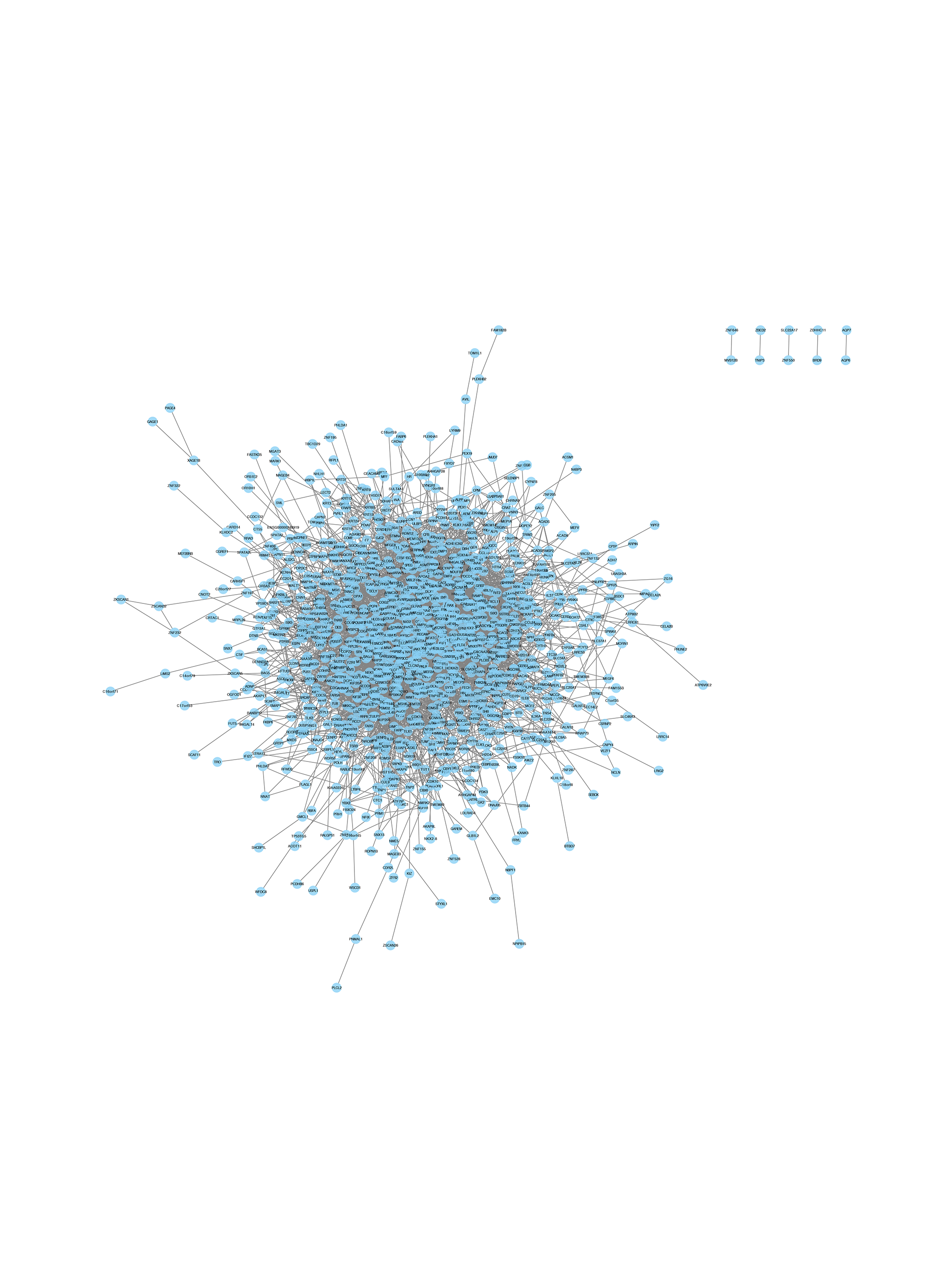


FigureS6. PPI network of module brown


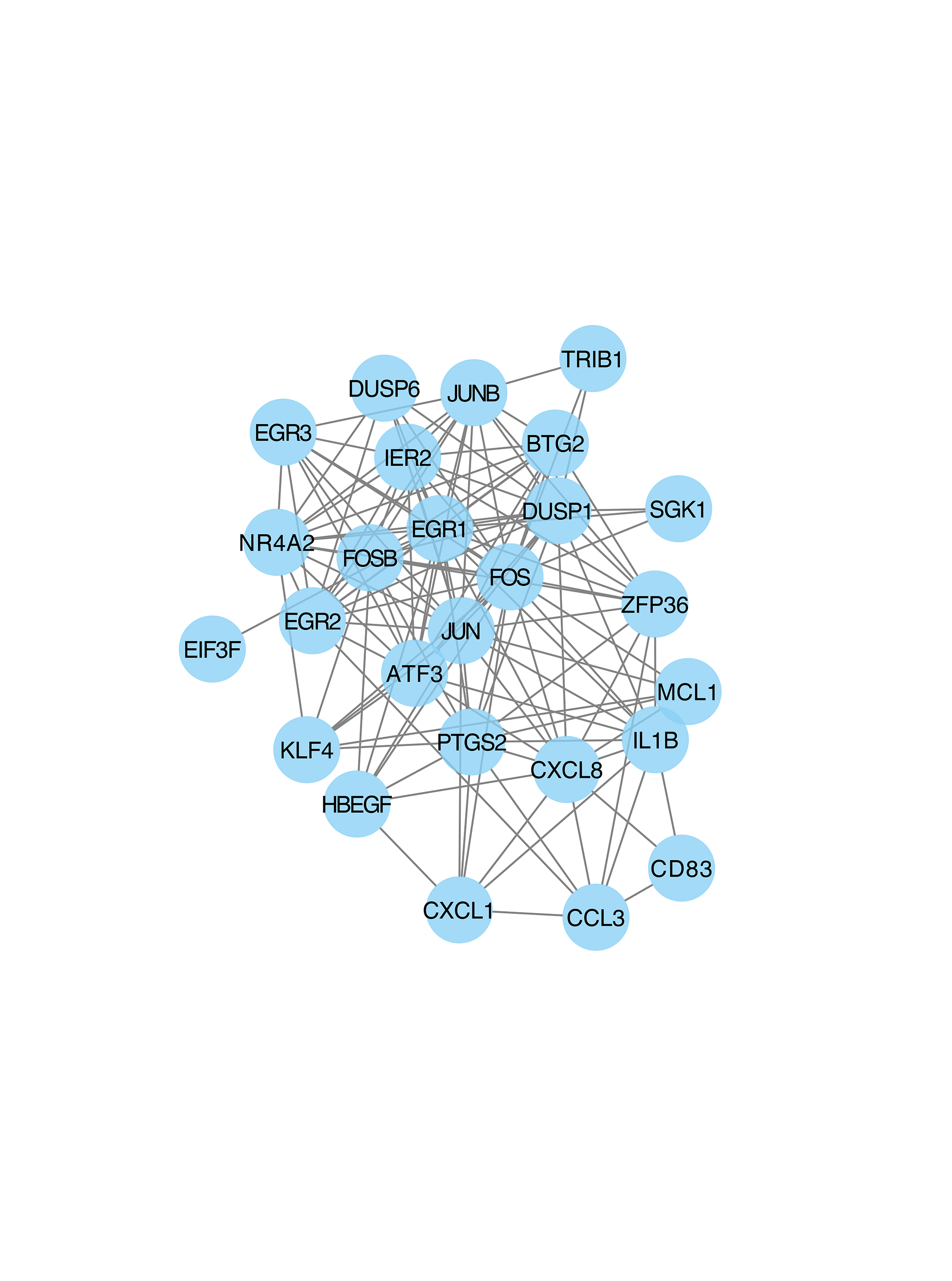


FigureS7. PPI network of module green yellow


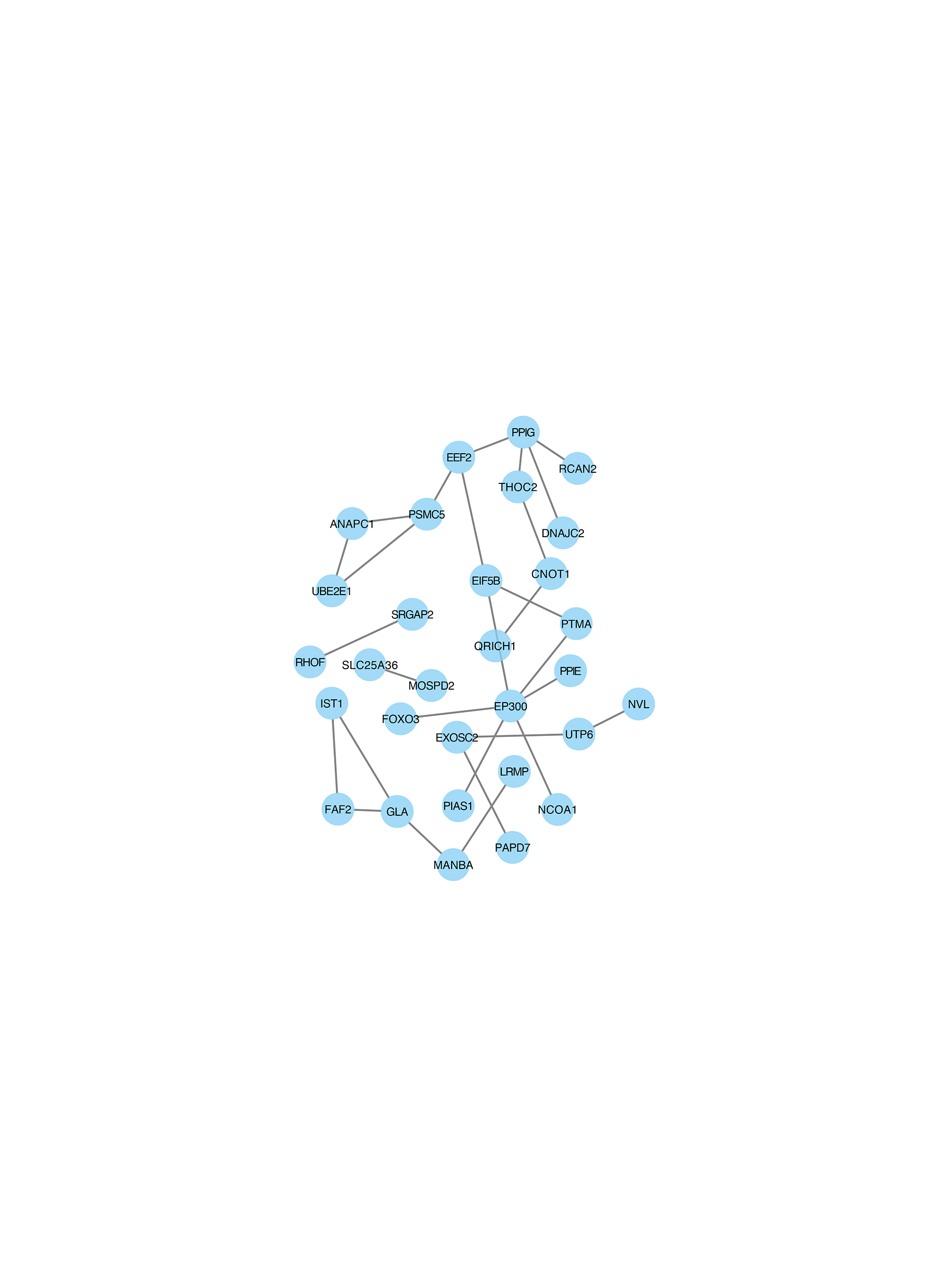


FigureS8. PPI network of module midnight blue


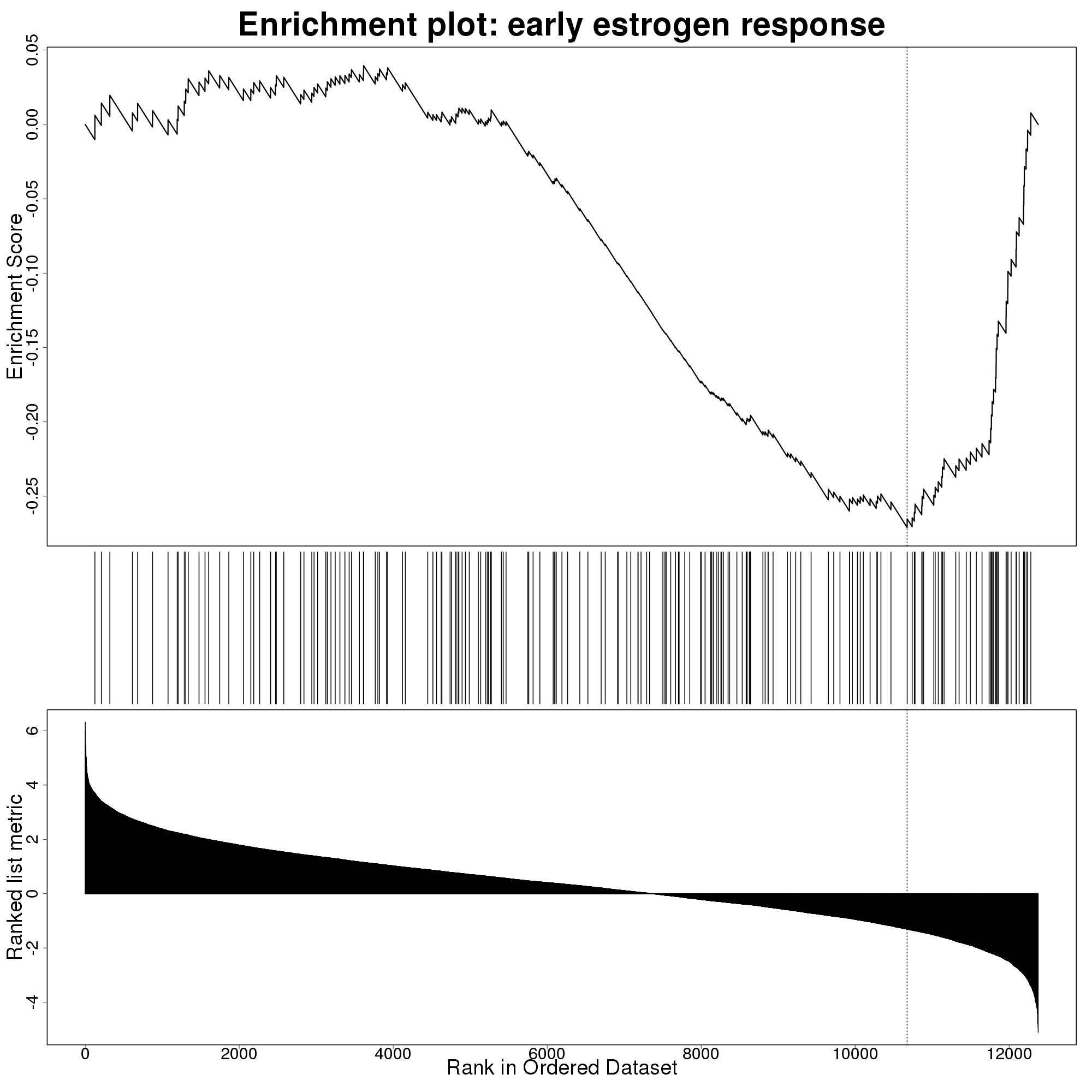


FigureS9. GSEA analysis for different expressed genes.


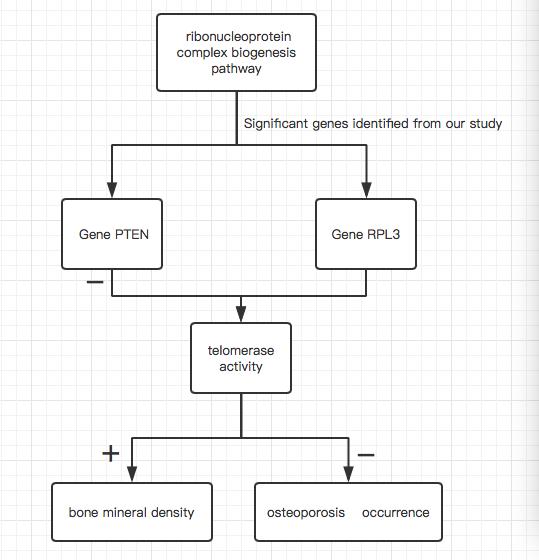


FigureS10. The schematic of relationship between ribonucleoprotein complex biogenesis and bone mineral density

FigureS11. The ROC of novel BMD score for predicting BMD in training data set 1

FigureS12. FigureS12. The ROC of novel BMD score for predicting BMD in test data set 1

FigureS13. The ROC of novel BMD score for predicting BMD in training data set 2

FigureS14. The ROC of novel BMD score for predicting BMD in test data set 2

FigureS15. The ROC of novel BMD score for predicting BMD in training data set 3

FigureS16. The ROC of novel BMD score for predicting BMD in test data set 3


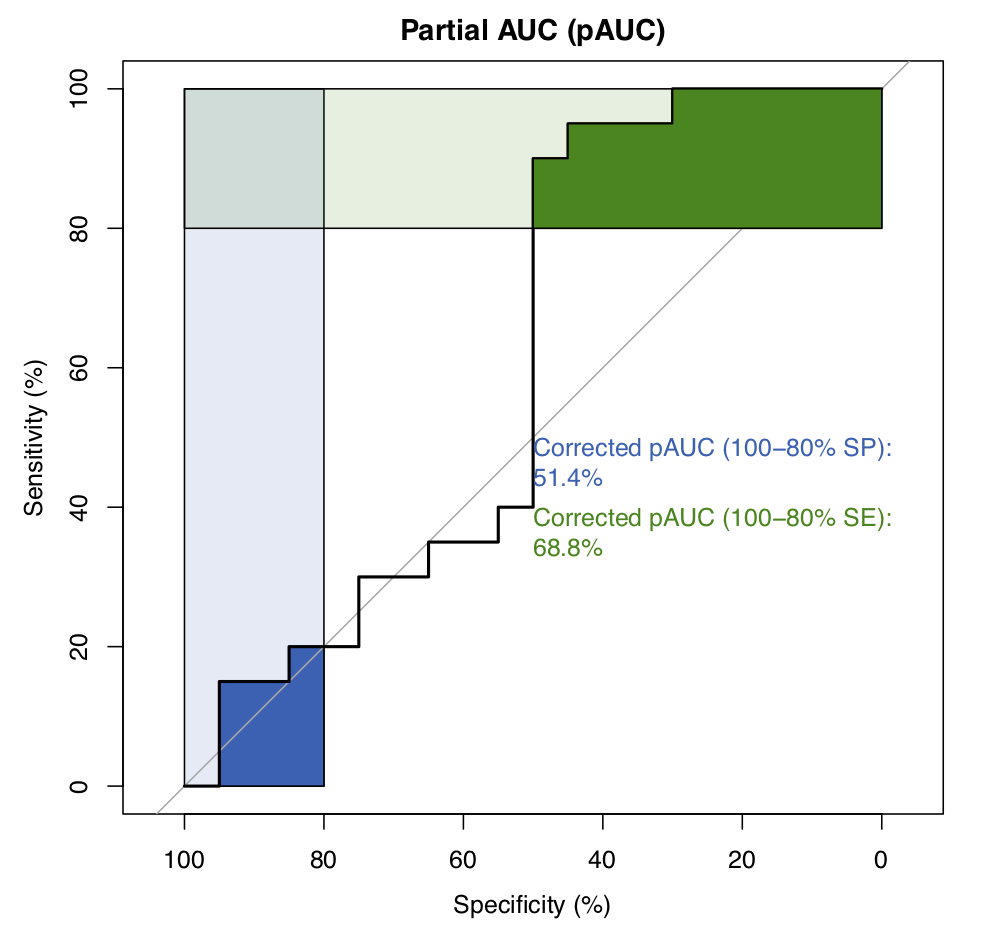


FigureS17. The ROC of novel BMD score for predicting BMD in test data set GSE13850


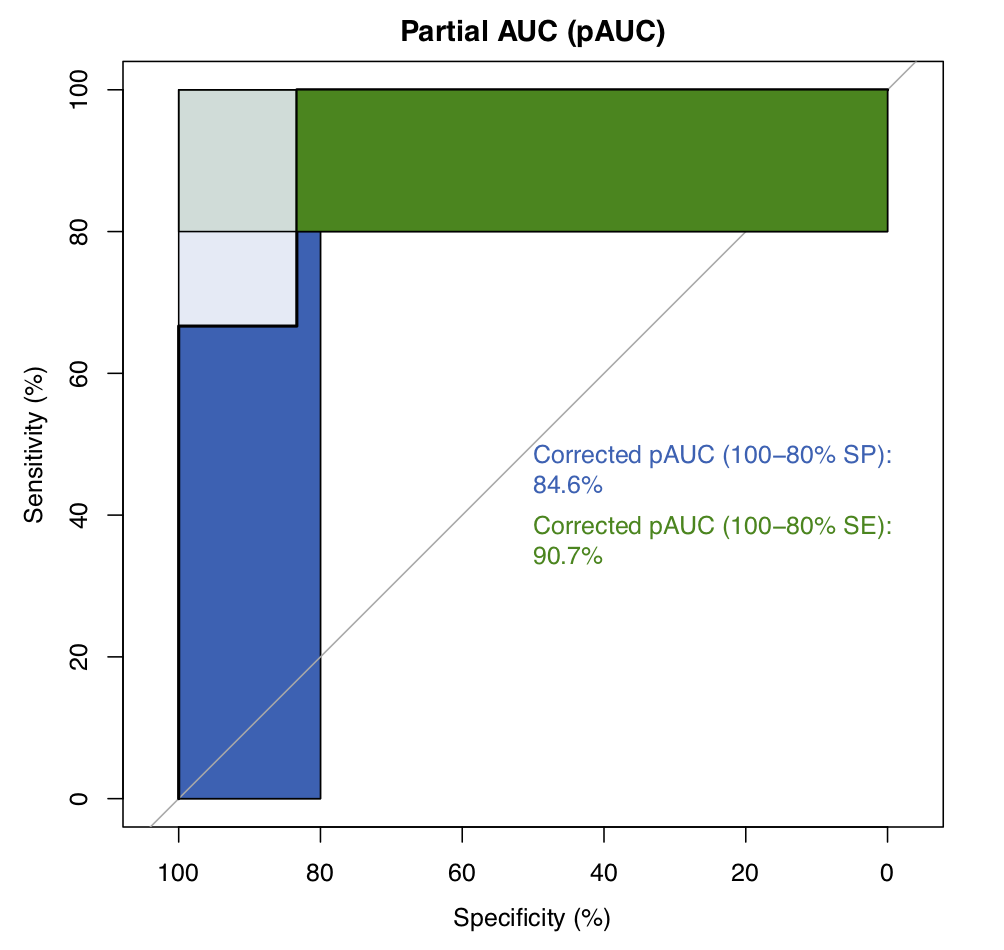


FigureS18. The ROC of novel BMD score for predicting BMD in test data set GSE20941

**Figure and table Legends**

FigureS1. Elastic net regression model

FigureS2. Power selection plot

FigureS3. Scatter plot of six modules. Scatter plots for salmon(A),Midnight blue(B), black(C), green yellow(D),brown(E), grey(F), respectively

FigureS4. PPI network of module salmon

FigureS5. PPI network of module black

FigureS6. PPI network of module brown

FigureS7. PPI network of module green yellow

FigureS8. PPI network of module midnight blue

FigureS9. GSEA analysis for different expressed genes

FigureS10. The schematic of relationship between ribonucleoprotein complex biogenesis and bone mineral density

FigureS11. The ROC of novel BMD score for predicting BMD in training data set 1

FigureS12. The ROC of novel BMD score for predicting BMD in test data set 1

FigureS13. The ROC of novel BMD score for predicting BMD in training data set 2

FigureS14. The ROC of novel BMD score for predicting BMD in test data set 2

FigureS15. The ROC of novel BMD score for predicting BMD in training data set 3

FigureS16. The ROC of novel BMD score for predicting BMD in test data set 3

FigureS17. The ROC of novel BMD score for predicting BMD in test data set GSE13850

FigureS18. The ROC of novel BMD score for predicting BMD in test data set GSE20941

Table S1.Functional enrichment analysis of genes in module black

Table S2.Functional enrichment analysis of genes in green yellow

Table S3.Functional enrichment analysis of genes in midnight blue

Table S4.Functional enrichment analysis of genes in sub network of module midnight blue

Table S5.Functional enrichment analysis of genes in module salmon

Table S6.Functional enrichment analysis of genes in sub network of module salmon

Table S7.Functional enrichment analysis of genes in module brown

Table S8.Functional enrichment analysis of genes in sub network 1 of module brown

Table S9.Functional enrichment analysis of genes in sub network 2 of module brown

Table S10.Functional enrichment analysis of genes in sub network 3 of module brown

Table S11.Functional enrichment analysis of genes in sub network 4 of module brown

Table S12.Functional enrichment analysis of genes in sub network 5 of module brown

Table S13.Functional enrichment analysis of genes in sub network 6 of module brown

Table S14.Detailed information about GSE13850 data set and GSE20941 data set
